# Association of BMI, Diet, Physical Activity, and Oral Hygiene Practices with DMFT Index of Male Dental Students at King Faisal University, Al-Ahsa

**DOI:** 10.3390/nu13010224

**Published:** 2021-01-14

**Authors:** Rizwan Jouhar, Muhammad Adeel Ahmed, Zohaib Khurshid, Syed Akhtar Hussain Bokhari

**Affiliations:** 1 Department of Restorative Dentistry and Endodontics, College of Dentistry, King Faisal University, Al-Ahsa 31982, Saudi Arabia; mshakeel@kfu.edu.sa; 2 Department of Prosthodontics and Dental Implantology, College of Dentistry, King Faisal University, Al-Ahsa 31982, Saudi Arabia; zsultan@kfu.edu.sa; 3 Department of Preventive Dental Sciences, College of Dentistry, King Faisal University, Al-Ahsa 31982, Saudi Arabia; sbokhari@kfu.edu.sa

**Keywords:** DMFT, oral hygiene practices, BMI, dietary habits, dental students, physical activity

## Abstract

Despite sufficient knowledge of good oral hygiene habits, dental students still suffer from oral health problems owing to dietary habits, obesity, and a sedentary lifestyle. This cross-sectional study was conducted to evaluate an association of BMI (body mass index), diet, physical activity, and oral hygiene practices with DMFT (decayed, missing, and filled teeth) of male dental students and interns at King Faisal University, Saudi Arabia, from August to September 2020. One hundred and eighty-five male participants completed the study questionnaire. The questionnaire consisted of sociodemographic information and nineteen close-ended questions about current diet, physical activity, and oral hygiene practices. Students’ height (cm) and weight (kg) were measured to calculate BMI. The principal investigator performed the oral clinical examinations for the DMFT status in the dental clinic. The chi-square test was used for the dichotomous variables and a *t*-test was used for the continuous variables. Linear and multinomial logistic regression were performed to detect the significant predictors of the DMFT score. The mean age of participants was 22.29 ± 2.13 years, and the BMI was 24.94 ± 3.36 (Kg/m^2^). Parents’ higher education and income levels were significantly (*p* < 0.001) associated with a higher BMI. Most dietary variables, especially sugar products, and low physical activity, were significantly (<0.047) associated with higher BMI. All oral hygiene practices, except miswak and mouthwash, were significantly (<0.003) associated with higher BMI. Decayed and missing teeth were significantly (<0.001) higher in the overweight and obese. A simple linear regression analysis demonstrated association between BMI and decayed teeth, with an R = 0.35 (<0.001); BMI and missing teeth had an R = 0.12 (*p* = 0.867); BMI and filled teeth had an R = 0.15 (*p* = 0.033), and BMI with DMFT had an R = 0.33 ((<0.001). This study demonstrated a strong significant association of the decayed and missing teeth with higher BMI levels. In addition, BMI was significantly associated with diet and physical activity, despite acceptable oral hygiene practices.

## 1. Introduction

Dental students are key members of society and are the future pillar of dentistry in Saudi Arabia. Despite having sufficient knowledge of good oral hygiene habits, they still suffer from oral health problems, owing to obesity, lifestyle, and possibly study pressure [1]. According to WHO, 1.9 billion adults with an age of 18 years and above, throughout the world, were found to be overweight in 2016, and out of these 650 million were obese. WHO defines overweight and obesity as “an abnormal or excessive fat accumulation that may impair health” [2]. Increasing prevalence of obesity is considered to be a significant public health problem in Saudi Arabia [3]. The primary cause of obesity is consumption of a high-calorie diet and a low calorie expenditure [4]. Additionally, physical inactivity and overweight are, respectively, found to be the fourth and fifth major causes of mortality worldwide, and are considered to be the leading risk factor for other systemic diseases like cardiovascular diseases, musculoskeletal diseases, and cancers of the liver, colon, and prostate [5].

Body Mass Index (BMI) is the most frequently used method to categorize people into underweight, normal weight, overweight, and obese [6]. Poor dietary habits, lack of effective oral hygiene practices, a sedentary lifestyle, and high sugar consumption, not only causes a deleterious effect on oral health but are also associated with obesity [3,7,8,9]. Young adults, including dental students, modify their lifestyle when they enter the university due to independence from their parents and changes in the social interaction [10]. Furthermore, most students feel stressful due to hectic schedules and workload, which in turn might influence them to adopt unhealthy lifestyle and dietary habits [1]. Previous studies reported that university students often neglect their healthy diet, oral hygiene practices, physical activity, and might start consuming junk food laden with excessive calories and tobacco products [11,12,13]. 

Dental caries is an infectious microbial disease of the teeth and the World Health Organization (WHO) found it to be one of the most prevalent diseases across the world, in 2017 [14]. Multiple factors are known to be involved in its prevalence, such as high frequency of sugar intake, poor oral hygiene, presence of cariogenic bacteria, and quality and quantity of saliva [15]. Presence of dental caries in the anterior teeth might compromise the esthetics and decrease the confidence level of dental students [16]. Moreover, pulpal pain and sensitivity owing to dental caries sometimes become unbearable and might affect the academic performance of dental students [17,18]. DMFT is a globally used index for assessing the status of dental health [19,20].

Dentists have an essential role in the diagnosis, prevention, and treatment of oral diseases and they contribute significantly to reduce the burden of oral diseases in the community. A persistent challenge for dental colleges is to produce future dentists who are equipped with thorough knowledge and skills, at par with worldwide excellence and competence [21,22,23,24,25]. Knowledge of good oral hygiene practices, healthy diet counselling, and frequent dental problems such as dental caries and tooth erosion are taught in the early academic years at dental schools, with the objective of students educating their patients during clinical practice. However, it was observed that knowledge and awareness could not produce the desired results until they were self-implemented and internalized. Therefore, it is desirable that future dentists should eat healthy food and maintain a good oral hygiene, in order to convince their patient to acquire a healthy lifestyle and adequate oral hygiene [26].

In light of this background, the present study aimed to evaluate an association of BMI (body mass index), diet, physical activity, and oral hygiene practices with DMFT (decayed, missing, and filled teeth) of male dental students and interns at King Faisal University, Al-Ahsa.

## 2. Materials and Methods

This cross-sectional study was carried out at College of Dentistry, King Faisal University, Al-Ahsa from August 2020 to September 2020. Study permission was attained from the committee of scientific research (KFU-REC/2020-11-01). The sample size was calculated using Open Epi, considering the mean DMFT level of 1.46 ± 2.04, with the power of study being 80. Keeping the confidence interval of 0.95% with an α error of 0.05%, the total estimated sample size was 190 participants [3]. 

One hundred and eighty-five male dental students participated after excluding 5-dropouts, owing to incomplete information provided in the questionnaire and withdrawal from participation. All participants belonged to the undergraduate dental programs, such as grade 2 (30), grade 3 (25), grade 4 (26), grade 5 (24), grade 6 (56), and Interns (24).

After obtaining written informed consent, a printed copy of the self-administered questionnaire was provided to the participants, who were advised to fill the required information. The reliability of the questionnaire was analyzed through Cronbach’s alpha. The split internal consistency of the questionnaire items was α = 0.704. Additionally, our research group checked the validity of the questionnaire through face and content validity methods in the panel discussion.

The questionnaire was divided into three sections. The first section contained sociodemographic information. The second section comprised of ten closed-ended questions to acquire information about current diet and physical activity. Oral hygiene practices of the participant were obtained by nine closed-ended questions in the third section.

After acquiring the required information in the questionnaire, each student’s height (cm) and weight (kg) was measured, and BMI was calculated using the National Heart, Lung and Blood Institute’s online BMI calculator (www.nhlbi.nih.gov). BMI score was classified into four categories of underweight (UW) (<18.5 Kg/m^2^), normal weight (NW) (18.5 to 24.9 Kg/m^2^), overweight (OW) (25 to 29.9 Kg/m^2^), and obese (Ob) (30 and above Kg/m^2^). All participants were in the scrub suit, and they were instructed to remove accessory wearing and shoes. Later, the predetermined weight of the scrub suit was subtracted from the determined weight.

Every participant underwent a thorough comprehensive clinical examination of the oral cavity. Primary teeth, early white spot lesion, and fissure sealant, were excluded. Teeth missing for reasons other than carious extraction, such as an orthodontic reason or trauma, were considered to be healthy, in line with the WHO criteria. All participants were allowed to sit comfortably on the dental chair (ESTETICA E30, KAVO, Warthausen, Germany). Visibility for dental examination were enhanced by using LED (KaVoLUX 540 LED, Biberach, Germany) attached to the dental chair. All teeth were air-dried using an air–water syringe, then cotton rolls were placed on the lingual and buccal sulcus for isolation. Basic examination instruments were used for the visual examination of the DMFT status.

DMFT score of all participants was assessed and recorded by a single examiner, the principal investigator, with intra-examiner agreement of 0.089 on the Kappa scale.

### Statistical Analysis

Statistical analysis was done on SPSS version 21. Tests of normality were applied to check the distribution of dental variables, such as Decayed, Missing, Filled Teeth (DMFT), Decayed Teeth (DT), Missing Teeth (MT), and Filled Teeth (FT) using the Kolmogorov test. Quantitative variables were analyzed as means and standard deviations, whereas qualitative variables were assessed in terms of frequencies and percentages. The chi-square test was used for dichotomous variables and control of covariates, i.e., the effect of independent variables BMI, diet, oral hygiene, physical activity on DMFT. A linear and multinomial logistic regression analysis was performed to detect significant predictors of the DMFT score. A *p*-value of ≤ 0.05 was considered to be significant.

## 3. Results

**Figure 1:**
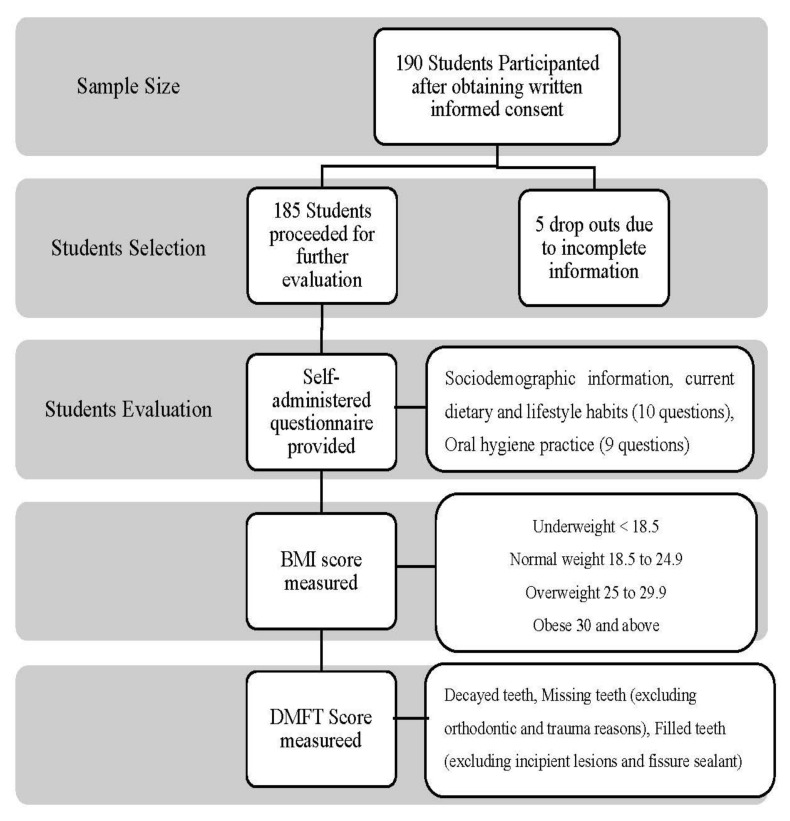
Flowchart of methodology.

One hundred and ninety (190) BDS (Bachelor’s in Dental Surgery) students, along with the interns, participated in the study. Data were analyzed for 185 participants, and five were eliminated due to incomplete information provided in the questionnaire (Figure 1). STROBE guidelines were followed in the study. Thirty students were from year 1, 25 from year 2, 26 from year 3, 24 from year 4, 56 from year 5, and 24 were internees. The mean age of participants was 22.29 ± 2.13 years, and BMI was 24.94 ± 3.36 Kg/m^2^.

Table 1 demonstrates the distribution of age, year of education, sibling, parent’s education, parent’s occupation, and family income, among the BMI categories of underweight, normal, overweight, and obese. Parents’ education levels and family’s income categories were statistically significant (*p* < 0.001) in the BMI categories.

Table 2 presents the distribution of dietary variables of meals per day, use of snacks in between meals, use of drinks, frequency of sweets, and use of free chewing gum. Most dietary variables were significantly (<0.047) distributed among the BMI categories except the use of eggs, orange juice, green tea with sugar and without sugar, other sweets, and use of chewing gum. Distribution of lifestyle variables of physical activity and entertainment activity during weekdays, weekends were significantly (*p* < 0.025) distributed among the BMI categories.

Table 3 explains the distribution of oral hygiene practices such as tooth brushing frequency per day, use of miswak, tooth-brushing technique, type of toothbrush, type of toothpaste, brush change time, use of interdental cleaning aids, use of mouthwash, and dental visits with respect to BMI categories. All variables showed significant (*p* < 0.003) distribution except the use of miswak and mouthwash.

**Figure 2:**
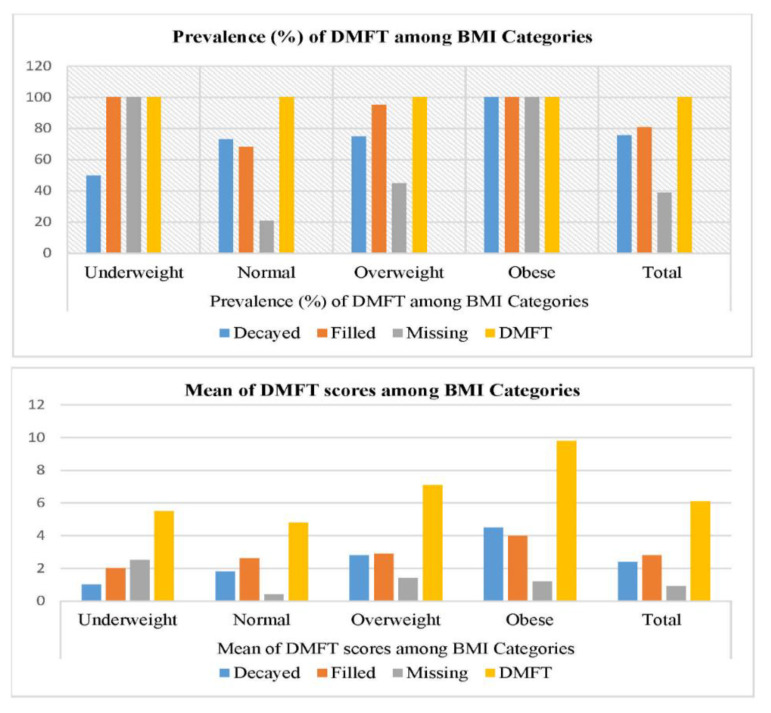
Status of DMFT and BMI categories among study participants.

Figure 2 explains the status of decayed teeth, missing teeth (excluding orthodontic and trauma reasons), filled teeth (excluding incipient lesions and fissure sealants) and DMFT, among BMI categories. Highest level of dental caries and filled teeth was noted in obese. In contrast, missing teeth were more in underweight students and the difference was highly significant (*p* < 0.001) for decayed and missing teeth. DMFT was highest in obese students, and the difference was highly significant (*p* < 0.001).

Table 4 demonstrates the association between BMI, dental caries, missing teeth, filled teeth, DMFT using simple linear regression analysis. Association of BMI and decayed teeth was explained by R = 0.35 (adjusted R^2^ 0.120, *p* = 0.000); BMI and missing teeth by R = 0.12 (adjusted R^2^ 0.07, *p* = 0.867); and BMI and filled teeth by R = 0.15 (adjusted R^2^0.01, *p* = 0.033). Association of BMI and DMFT was explained by R = 0.33 (adjusted R^2^ 0.10, *p* = 0.000).

There was a significant difference (*p* <0.01) when the covariates (i.e., age, BMI, diet, oral hygiene, and physical activity) were analyzed as confounders with the DMFT index in participants.

Table 5 demonstrates the association between the BMI categories (1 = underweight and normal weight, 2 = overweight and obese), tooth brushing timing, type of toothbrush, use of fluoridated toothpaste, and use of dental floss and dental caries (yes/no), missing teeth (yes/no), and filled teeth (yes/no). A significant association was shown by BMI categories with missing teeth (*p* < 0.001) and filled teeth (*p* < 0.001); toothbrush type categories with decay (*p* < 0.001), and interdental cleaning categories with decayed teeth (*p* < 0.001) and missing teeth (*p* < 0.001). Status of dental caries (yes/no), missing teeth (yes/no), filled teeth (yes/no) was further assessed for association with socioeconomic status, frequency of use of sweets, and moderate physical activity. Decayed teeth were significantly associated with father’s education (*p* < 0.001), family income (*p* < 0.001), use of candy (*p* < 0.001) and dates (*p* < 0.001), weekend physical activity (*p* < 0.050), and weekday TV and video game use (*p* < 0.050).

Missing teeth were significantly associated with mother’s occupation (*p* < 0.050), use of candy (*p* < 0.001), weekday physical activity (*p* < 0.050). Filled teeth were significantly associated with mother’s occupation (*p* < 0.050), and weekday TV and video game use (*p* < 0.050).

## 4. Discussion

Obesity and dental caries have multi-factorial etiologies with a common contributing factor to their occurrence, i.e., dietary habits and food intake [27]. Dental caries were declared a non-communicable disease of concern worldwide, and it was the most prevalent disease condition to be included in the 2015 Global Burden of Disease study [14,28,29]. The literature gives conflicting pieces of evidence regarding the relationship between patient weight and the status of their oral health and hygiene [30]. On the one hand, there are studies that demonstrated the association of incidence of a high number of caries rates in overweight (OW) and obese (O) subjects [31,32]. Despite this, there are studies that claim that no association whatsoever exists between BMI and dental caries, or even periodontitis, for that matter [33,34,35].

Nonetheless, the purpose of our study was to assess the association between body mass index (BMI), dietary habits, and oral hygiene practices, based on DMFT index among male dental students at King Faisal University, Al Ahsa. Out of the 185 participants in this study; 6 (3.2%) were underweight (UW), 101 (54.6%) were of normal weight (NW), 60 (32.4%) were overweight, and 18 (9.7%) were obese.

The BMI of this study sample averaged 24.94 ± 3.36 kg/m^2^. According to the BMI categories, the majority of our sample was nearing the overweight aspect of the BMI spectrum. The trend was supported by a study by Al-Shehri et al. [1], who demonstrated that university students on the brink of being young adults entering the practical world tend to make some drastic lifestyle changes, which could negatively impact their oral health, as well as general health and well-being. Based on the National Nutrition Survey 2007 [1,36], the Kingdom of Saudi Arabia had more obese females (23.6%) as compared to males (14.2%), with 30.7% overweight males and 28.4% overweight females; an alarming situation for a country to have more than 50% of the population in the overweight/obese categorization, with our study results somewhat matching the survey outcomes.

The education levels of parents and family income and sociodemographic variables showed a significantly high relationship with BMI categories. About 50% (*n* = 9) of obese students had fathers with school-level education, and 45% (*n* = 27) of the overweight students had fathers with university-level education (*p* = <0.001). Likewise, 83.3% (*n* = 15) of the obese students had mothers with school-level education (*p* = <0.001). A cross-sectional survey conducted by Al-Agha et al. [37] in Jeddah, Saudi Arabia, concluded that BMI levels increased with decrease in educational level of parents, consistent with the findings in our study. Similarly, a cross-sectional survey conducted on obese children in Jeddah also condoned the fact that education levels of parents, especially mothers, play a crucial role in the dietary habits and lifestyles of children. As mothers spend more time with their children as compared to fathers, less educational years on their part usually result in children with the highest risks of being obese [38].

The presence of family is imperative in shaping the dietary habits, eating, and physical activity patterns of the members in the construct [39]. Fathers with government jobs in our study had a far greater likelihood of having overweight (*n* = 36, 60%) and obese (*n* = 15, 83.3%) children (*p* = 0.013). The role of income showed that 100% of obese participants (*n* = 18) and 55% (*n* = 33) overweight participants had a cumulative family income between SAR 10,001–20,000 (*p* = 0.001). A systematic review and meta-analysis conducted by Kim et al. in 2018 [40] corroborated these findings, with an OR of 1.27 indicating a higher chance and a relative risk of 1.52 for obesity occurring in low-income groups. The meta-analysis concluded that those individuals who were on the lower-income scale of socio-demographics were more likely to be obese than their non-obese counterparts. Data substantiated by Asiseh et al. [41] from China also verified our findings, with results claiming that middle-class groups were the ones at the highest risk of being overweight and obese among other income categories.

An overload of free sugars and frequent meals heavy on calories, with physical inactivity, subsequently increased BMI, [1] poor oral health, and incidence of dental caries [14,29]. The assessed dietary variables in our study showed a significant association with BMI overweight and obese categories. Consumption of nuts as a snack in between meals was seen in 55.4% (*n* = 56) of normal-weight participants, 60% (*n* = 36) of overweight, and 83.3% (*n* = 15) of obese participants in our study (*p* = 0.004). Although the amount of consumption of some foods and drinks were not assessed in our study, nuts contain high calories, which could explain the consumption among the overweight/obese participants. However, the long-term benefit of consuming a regular one handful of nuts was associated with lower weight gain [42]. Intake of chocolate was consistently observed in normal-weight (*n* = 83, 82.2%), overweight (*n* = 48, 80%), and obese (*n* = 18, 100%) participants, which was significantly distributed (*p* = 0.040), as was the intake of ice cream (NW: *n* = 57, 56.4%, OW: *n* = 36, 60%, Ob: *n* = 18, 100%, *p* = 0.005), candy (OW: *n* = 42, 70%, Ob: *n* = 18, 100%, *p* = 0.000), soft drinks (NW: *n* = 47, 46.5%, OW: *n* = 51, 85%, Ob: *n* = 15, 83.3%, *p* = 0.000), and chips (NW: *n* = 50, 49.5%, OW: *n* = 33, 55%, Ob: *n* = 18, 100%, *p* = 0.001). Moreover, all obese participants had a habit of holding drinks in their mouth (*p* = 0.000). These deleterious habits and poor food choices were observed amongst the overweight and obese participants, as was demonstrated by a number of studies in the literature [1,26,43,44].

Physical activity, both on weekends and weekdays, was consistently and significantly distributed for the respective BMI categories (Table 3). Students tended to kick back and relax during weekends, indulging in leisurely activities like playing video games and watching television, for usually more than 30 min. Physical activity has an inverse relationship with being overweight and obese, as was concluded by Ahmed et al. [45] Moreover, our study showed significant findings in the DMFT index in relation to frequent use of sweets, socioeconomic status, and moderate physical activity. This was in accordance with Ferrazzano et al. [46] who reported a high risk of dental caries in children with high consumption of sugary food. They also found a higher DMFT score in children with less parental guidance and highlighted the significant role of parents in oral health education of their children.

Oral health and hygiene maintenance in our study was evaluated by the use of the DMFT index. Dental caries was significantly higher in overweight (2.80 ± 2.39) and obese (4.50 ± 0.985) participants (*p* = 0.000) (Table 5). As opposed to the results reported by Idrees et al. [4], who claimed that no significant correlation was found between the severity of dental caries and BMI categories. Contrarily, Bhayat et al. [3] in their study concluded with the finding that boys in the underweight and normal BMI groups were more prone to developing caries (*p* < 0.05), as compared to overweight and obese.

Over the years, conflicting studies accumulated regarding the relationship between dental caries and BMI. Systematic reviews and meta-analyses [47,48,49] tried to find out a tangible proof regarding the nature of the association between the two variables. Among the included studies in the systematic review conducted by Paisi et al. [47], 26 studies showed a positive correlation between dental caries and BMI, 19 demonstrated a negative association, 43 did not show any association. In contrast, some studies showed surprisingly more than one pattern of association between the variables of interest. Alghamdi et al. [50] reported statistically negative association between BMI and dental caries. This difference in result might be due to frequent snacking with relatively high amount of refined sugar among underweight children, which in turn suppress their desire to consume main meal. Thus, eventually resulting in high incidence of dental caries.

In addition, our study also reported significant association of BMI categories with respect to the brushing technique used (*p* < 0.001), type of toothpaste (*p* = 0.003), type of toothbrush (*p* < 0.001), brush change time (*p* = 0.001), and the interdental cleaning aids (*p* < 0.001). Moradi et al. [19] also showed significant findings regarding the use of toothbrush, mouthwash, and dental floss. In their study, participants who used mouthwashes and brushed their teeth along with regularly using dental floss, were less likely to have decayed, missing, and filled teeth, as assessed by the DMFT index.

This study demonstrated highest status of correlation between the DMFT index in obese students (*p* < 0.001), with dental caries manifesting the most in overweight (DT= 2.80 ± 2.39) and obese (DT= 4.50 ± 0.985) participants. Furthermore, the association of BMI with decayed teeth (*p* < 0.001), filled teeth (0.033), and DMFT index (*p* < 0.001) were highly significant, when adjusted for confounders in the linear regression model. These findings were corroborated by two studies conducted in India [51,52], which also culminated in positive associations between BMI and dental caries. Sakeenabi et al. [51] stated that among the 6-years old children in their study cohort who were either overweight or obese, 1.92- and 3.6-times had a higher risk of developing dental caries, respectively. They further stated that overweight and obese children who were 13-years old in their cohort had 1.68 and 1.8 times the risk of developing dental caries, compared to their normal weighted counterparts.

## 5. Limitations

Even though our results showed some positive outcomes regarding the variables of interest, no study is without weaknesses and limitations. Our study included males as participants, because of the relatively strict segregation of males and females, and particular cultural sensitivities in the Kingdom of Saudi Arabia. Thus, our findings cannot be applied to the opposite gender. Cross-sectional studies and convenience sampling techniques are inherent of the lower evidence-based spectrum; hence causal inferences cannot be established between the identified variables [27]. Dental caries were determined by visual examination, and x-rays were not taken to verify the diagnosis.

## 6. Conclusions

This study further augmented the contributory role of diet in obesity and dental caries. A strong and significant relationship of decayed and missing teeth with higher BMI levels was shown. In addition, despite having acceptable oral hygiene practices, BMI also revealed a significant association with diet and physical activity.

## Figures and Tables

**Table 1:** Distribution of sociodemographic variables [*n* (%)/(Mean ± SD)] among the BMI Categories.

**Variables**	**BMI Categories (Kg/m^2^)**					
	**Underweight**	**Normal**	**Overweight**	**Obese**	**Total**	***p*-Value**
	6 (3.2)	101 (54.6)	60 (32.4)	18 (9.7)	185 (100)	
	17.83 ± 0.41	22.73 ± 1.31	27.38 ± 0.98	31.61 ± 1.11	24.94 ± 3.36	<0.001
**Age (Years)**	22.33 ± 2.58	22.28 ± 2.03	22.38 ± 2.28	22.29 ± 2.13	22.29 ± 2.13	0.931
Age group 1 (19–22 years)	3 (50)	50 (49.5)	27 (45)	11 (61)	91 (49.2)	0.694
Age group 2 (23–26 years)	3 (50)	51 (50.5)	33 (55)	7 (38.9)	94 (50.8)	
**BDS Year of Education**						
Year 1	1 (16.7)	15 (14.9)	10 (16.7)	4 (22.2)	30 (16.2)	0.827
Year 2	1 (16.7)	14 (13.9)	9 (15.0)	1 (5.6)	25 (13.5)	
Year 3	0	15 (14.9)	6 (10.0)	5 (27.8)	26 (14.1)	
Year 4	2 (33.3)	15 (14.9)	6 (10.0)	1 (5.6)	24 (13.0)	
Year 5	1 (16.7)	30 (29.7)	20 (33.3)	5 (27.8)	56 (30.3)	
Internees	1 (16.7)	12 (11.9)	9 (15.0)	2 (11.1)	24 (13)	
**Sibling**						
5/1	4 (66.7)	60 (59.4)	34 (56.7)	8 (44.4)	106 (57.3)	0.216
10/6	1 (16.7)	39 (38.6)	22 (36.7)	10 (55.6)	72 (38.9)	
>10	1 (16.7)	2 (2.0)	4 (6.7)	0	7 (3.8)	
**Father Education**						
No Education	3 (50)	27 (26.7)	3 (5.0)	0	33 (17.8)	<0.001
School level	0	9 (8.9)	15 (25)	9 (50)	33 (17.8)	
College level	0	17 (16.8)	15 (25)	6 (33.3)	38 (20.5)	
University level	3 (50)	48 (47.5)	27 (45)	3 (16.7)	81 (43.8)	
**Mother Education**						
No Education	3 (50)	36 (35.6)	21 (35)	0	60 (32.4)	<0.001
School level	3 (50)	21 (20.8)	21 (35)	15 (83.3)	60 (32.4)	
College level	0	9 (8.9)	0	0	9 (4.9)	
University level	0	35 (34.7)	18 (30)	3 (16.7)	56 (30.3)	
**Father Occupation**						
Government Service	6 (100)	62 (61.4)	36 (60)	15 (83.3)	119 (64.3)	0.013
Private Service	0	15 (25.0)	15 (25)	0	30 (16.2)	
Business	0	15 (14.9)	9 (15)	0	24 (13.0)	
Others	0	9 (8.9)	0	3 (16.7)	12 (6.5)	
**Mother Occupation**						
Government Service	0	29 (28.7)	12 (20)	0	41 (22.2)	0.077
Private Service	0	3 (3)	3 (5)	0	6 (3.2)	
Business	0	0	0	0	0	
Others (house wife)	6 (100)	69 (68.3)	45 (75)	18 (100)	138 (74.6)	
**Family Income**						
<10,000	3 (50)	27 (26.7)	12 (20)	0	42 (22.7)	
10,001–20,000	3 (50)	42 (41.6)	33 (55)	18 (100)	96 (51.9)	0.001
20,000–30,000	0	26 (25.7)	9 (15)	0	35 (18.9)	
>30,000	0	6 (5.9)	6 (10)	0	12 (6.5)	

**Table 2:** Distribution of Diet and Physical Activity variables [*n* (%)/(Mean ± SD) ] among the BMI Categories.

	**BMI Categories (Kg/m^2^)**				
**Variables**	**Underweight**	**Normal**	**Overweight**	**Obese**	***p*-Value**
	6 (3.2)	101 (54.6)	60 (32.4)	18 (9.7)	
**Frequency of main meals**					
1 meal /day	0	12 (11.9)	3 (5)	0	0.034
2 meals/day	0	41 (40.6)	18 (30)	6 (33.3)	
3 meals / day	6 (100)	48 (47.5)	36 (60)	12 (66.7)	
>3 meals/day	0	0	3 (5)	0	
**Use of Snacks in between meals**	6 (100)	98 (97)	60 (100)	15(83.3)	0.006
Raw vegetables	3 (50)	63 (62.4)	27 (45)	6(33.3)	0.048
Fruits	6 (100)	77 (76.2)	48 (80)	12 (66.7)	0.347
Nuts	0	56 (55.4)	36 (60)	15 (83.3)	0.004
Eggs	3 (50)	48 (47.5)	36 (36.4)	12 (66.7)	0.289
Yogurt plain	3 (50)	51 (50.5)	42 (70)	15 (83.3)	0.014
Yogurt fruit	6 (100)	24 (23.8)	9 (15)	0	0.047
Chocolate	3 (50)	83 (82.2)	48 (80)	18 (100)	0.04
Smoothies	0	42 (41.6)	15 (25)	9 (50)	0.024
Cheese	3 (50)	48 (47.5)	42 (70)	12 (66.7)	0.035
Ice cream	3 (50)	57 (56.4)	36 (60)	18 (100)	0.005
Popcorn	0	36 (35.6)	21 (35)	3 (16.7)	0.134
Candy	0	30 (29.7)	42 (70)	18 (100)	0
Soft drink	0	50 (49.5)	48 (80)	18 (100)	0
Juice	6 (100)	65 (64.4)	42 (70)	15 (83.3)	0.139
Chips	3 (50)	50 (49.5)	33 (55)	18 (100)	0.001
**Use of Drinks**					
Lemon Juice	3 (50)	42 (41.6)	33 (55)	3 (16.7)	0.033
Orange Juice	0	9 (8.9)	6 (10)	3 (16.7)	0.634
Green tea with sugar	0	9 (8.9)	9 (15)	3 (16.7)	0.446
Green tea without sugar	0	29 (28.7)	12 (20)	3 (16.7)	0.24
Black Coffee with sugar	0	3 (3)	15(25)	3 (16.7)	0
Black Coffee without sugar	3 (50)	62 (61.4)	24 (40)	12 (66.7)	0.043
Arabic tea non-sugary	0	59 (58.4)	30 (50)	9 (50)	0.041
Soft drinks	0	47 (46.5)	51 (85)	15 (83.3)	0
Hold drink in mouth (yes)	6 (100)	53 (52.3)	21 (35)	18 (100)	0
Hold for <1 min	3 (50)	53 (52.3)	21 (35)	3 (16.7)	0.015
Hold for >1 min	3 (50)	48 (47.5)	39 (65)	15 (83.3)	
**Frequency of Sweets**					
Chocolate bar	3 (50)	77 (76.2)	54 (90)	18 (100)	0.004
Candy	3 (50)	36 (35.6)	42 (70)	18 (100)	0
Dates	6 (100)	77 (76.5)	51 (85)	18 (100)	0.047
Other sweets	0	3 (3)	6 (10)	3 (16.7)	0.08
**Physical Activity on week days**					
None	3 (50)	6 (5.9)	18 (30)	12 (66.7)	0
<30 min	0	24 (23.8)	18 (30)	6 (30.3)	
>30 min	3 (50)	71 (70.3)	24 (40)	0	
**Physical Activity on week end**					
None	0	3 (3)	18 (30)	3 (16.7)	0
<30 min	3 (50)	18 (17.8)	12 (20)	9 (50)	
>30 min	3 (50)	80 (79.2)	30 (50)	6 (33.3)	
**Watch TV, Play Video games on week days**					
None	0	23 (22.8)	6 (10)	3 (16.7)	0.025
<30 min	3 (50)	24 (23.8)	12 (20)	15 (83.3)	
>30 min	3 (50)	54 (53.5)	42 (70)	0	
**Watch TV, Play Video games on week end**					
None	0	14 (13.9)	0	0	0.02
<30 min	0	21 (20.8)	12 (20)	3 (16.7)	
>30 min	6 (100)	66 (65.3)	48 (80)	15 (83.3)	
Use Sugar free chew gum	3 (50)	44 (43.6)	36 (60)	9 (50)	0.254

**Table 3:** Distribution of Oral Hygiene Practices [*n* (%)] among BMI Categories.

	**BMI Categories (Kg/m^2^)**				
**Variables**	**Underweight**	**Normal**	**Overweight**	**Obese**	***p*-Value**
	6 (3.2)	101 (54.6)	60 (32.4)	18 (9.7)	
**Tooth Brushing**					
None	0	0	3 (5)	0	0
Once daily	0	23 (22.8)	36 (60)	15 (83.3)	
Twice daily	3 (50)	63 (62.4)	18 (30)	3 (16.7)	
>Twice daily	3 (50)	15 (14.9)	3 (5)	0	
**Miswak**					
None	6	57 (56.4)	39 (65)	9 (50)	0.242
Daily	0	3 (3)	3 (5)	0	
Occasionally	0	41 (40.6)	18 (30)	9 (50)	
**Brushing Technique**					
Bass	0	0	6 (10)	0	0
Modified Bass	6 (100)	95 (94.1)	36 (60)	12 (66.7)	
Stillman	0	0	3 (5)	0	
Modified Stillman	0	0	9 (15)	0	
Charters	0	3 (3)	3 (5)	3 (16.7)	
Rolling	0	3 (3)	3 (3)	0	
Horizontal	0	0	0	3 (16.70	
**Type of Toothpaste**					
Fluoridated	6 (100)	92 (91.1)	48 (80)	12 (66.7)	0.003
Non-fluoridated	0	3 (3)	0	0	
Anti-sensitive	0	3 (3)	3 (5)	0	
Others	0	0	3 (5)	0	
Do not know	0	3 (3)	6 (10)	6 (33.3)	
**Type of Tooth brush**					
Extra-soft	3 (50)	15 (14.9)	9 (15)	0	0
Soft	3 (50)	45 (44.6)	39 (65)	3 (16.7)	
Medium	0	35 (34.7)	12 (20)	15 (83.3)	
Electric	0	6 (5.9)	0	0	
**Brush Change time**					
Once in 3 months	6 (100)	50 (45)	27 (45)	6 (33.3)	0.001
Once in 6 months	0	45 (44.6)	30 (50)	6 (33.3)	
Once in year	0	6 (5.9)	3 (5)	6 (66.3)	
**Interdental Cleaning Aid Name**					
Flossing	3 (50)	18 (17.8)	15 (25)	18 (100)	0
Wooden toothpick	0	44 (43.6)	21 (35)	0	
Plastic toothpick with floss	0	6 (5.9)	6 (10)	0	
Water-pick	0	6 (5.9)	3 (5)	0	
Interdental brush	3 (50)	27 (26.7)	15 (25)	0	
**Use Mouthwash**					
None	3 (50)	42 (41.6)	24 (40)	6 (33.3)	0.415
Once daily	0	15 (14.9)	6 (10)	0	
Occasionally	3 (50)	44 (43.6)	30 (50)	12 (66.7)	
**Dental Visit**					
Every 3 months	0	12 (11.9)	3 (5)	0	0
Every 6 months	6 (100)	42 (41.6)	15 (25)	0	
Every year	0	20 (19.8)	18 (30)	3 (16.7)	
Only when required	0	24 (23.8)	24 (40)	15 (83.3)	
Never	0	3 (3)	0	0	

**Table 4:** Association of BMI with number of decayed teeth, missing teeth, filled teeth, and DMFT *.

Model	Unstandardized Coefficients		Standardized Coefficients	t	Sig.	95.0% Confidence Interval for B	
	B	Std. Error	Beta			Lower Bound	Upper Bound
BMI & Decayed teeth	0.228	0.045	0.353	5.112	0.000	0.140	0.315
BMI & Missing Teeth	0.049	0.032	0.112	1.523	0.676	0.014	0.112
BMI & Filled Teeth	0.118	0.055	0.157	2.154	0.033	0.010	0.225
BMI & DMFT	0.394	0.082	0.336	4.822	0.000	0.233	0.555

* Simple linear regression analysis, using decayed, missing, filled teeth, and DMFT, as dependent variables.

**Table 5:** Association between BMI, tooth brushing time, toothbrush type, use of fluoridated toothpaste, interdental cleaning and decayed (yes/no), missing (yes/no), and filled teeth (yes/no) categories.

	Decayed (Yes/No)		Missing (Yes/No)		Filled (Yes/No)	
Variables	*n*	Adjusted OR	*n*	Adjusted OR	*n*	Adjusted OR
		(C.I. 95%)		(C.I. 95%)		(C.I. 95%)
**BMI**						
Underweight and Normal weight ^#^	77/30	1.41(0.5–3.50)	27/80	3.99(1.80–8.82) **	75/32	9.23(2.30–37.03) **
Overweight and Obese	63/15		45/33		75/3	
**Toothbrush time**						
1 time daily ^#^	65/12	0.90(0.3–2.30)	39/38	1.23(0.54–2.81)	69/8	0.75(0.26–2.14)
≥2 times daily	75/33		33/75		81/27	
**Toothbrush type**						
Extra or soft ^#^	78/39	6.05(2.1–16.9) **	45/72	1.06(0.49–2.26)	99/18	0.48(0.20–1.14)
Medium or hard	62/6		27/41		51/17	
**Fluoridated toothpaste**						
Yes ^#^	119/42	0.17(0.02–0.10)	60/101	0.38(0.13–1.13)	129/32	0.42(0.07–2.30)
Other	21/3		12/12		21/3	
**Interdental cleaning**						
Dental floss ^#^	63/3	0.14(0.01–0.25) **	42/24	0.17(0.07–0.37) **	60/6	0.33(0.10–1.10)
Other	77/42		30/89		90/29	
**Socioeconomic Status**						
Father Education (Yes) ^#^	122/30	3.38(1.53–7.79) *	60/92	1.14(0.05–2.49)	126/26	1.81(0.75–4.33)
Mother Education (Yes) ^#^	92/33	0.89(0.68–1.16)	45/80	6.68(0.36–1.28)	99/26	0.67(0.29–1.54)
Father Occupation (Yes) ^#^	140/45	0.92(0.65–1.31)	72/113	1.18(0.86–1.61)	150/35	0.73(0.51–1.05)
Mother Occupation (Yes) ^#^	32/15	0.59(0.28–1.23)	9/38	0.28(0.17–0.62) **	33/14	0.45(0.19–0.92) **
Family Income (>10,000) ^#^	116/27	3.22(1.53–6.75) *	54/89	0.80(0.40–1.62)	120/23	2.08(0.93–4.68)
**Frequency of Sweets**						
Chocolate bar (Yes) ^#^	116/36	1.20(0.51–2.83)	60/92	1.14(0.52–2.49)	120/32	0.37(0.10–1.30)
Candy (Yes) ^#^	78/12	3.46(1.65–7.25) **	54/36	6.14(3.30–12.46) *	78/12	2.07(0.96–4.46)
Dates (Yes) ^#^	125/27	5.55(2.49–12.30) **	60/92	1.10(0.50–2.10)	132/20	5.50(2.39–12.62)
Other sweets (Yes) ^#^	9/3	0.96(0.24–3.71)	6/6	1.62(0.50–5.23)	12/10	----
**Physical Activity (Moderate Level)**						
On weekdays (Yes) ^#^	110/36	0.91(0.39–2.10)	45/101	0.19(0.09–0.42) **	114/32	0.29(0.08–1.02)
On weekend (Yes) ^#^	128/33	3.8(1.59–9.41) **	63/92	1.07(0.44–2.59)	129/32	0.57(0.16–2.05)
**Watch TV play videogames**						
On weekdays (Yes) ^#^	111/42	0.27(0.07–0.94) **	69/84	7.94(2.32–27.80)	129/24	2.81(1.20–6.58) **
On weekend (Yes) ^#^	126/45	0.15(0.05–0.74)	72/99	0.21(0.11–0.38)	138/33	0.69(0.14–3.24)

Logistic regression analysis. ^#^ Reference, C.I. —confidence interval, * *p* < 0.05, ** *p* < 0.001.

## Data Availability

The data presented in this study are available on request from the corresponding author. The data are not publicly available due to ethical concerns.
